# Leaving no stone unturned: the search for stroke associated with atrial fibrillation

**DOI:** 10.1002/acn3.51651

**Published:** 2022-08-30

**Authors:** Ana Catarina Fonseca

**Affiliations:** ^1^ Stroke Unit, Department of Neurology, Hospital de Santa Maria Centro Hospitalar Universitário Lisboa Norte Lisbon Portugal; ^2^ Cerebral Hemodynamic Lab, Hospital de Santa Maria Centro Hospitalar Universitário Lisboa Norte Lisbon Portugal; ^3^ Instituto de Medicina Molecular, Faculdade de Medicina Universidade de Lisboa Lisbon Portugal; ^4^ Centro de Estudos Egas Moniz, Faculdade de Medicina Universidade de Lisboa Lisbon Portugal

The diagnosis of paroxysmal atrial fibrillation (AF) continues to be a main priority in stroke patients. After the clinical trials that evaluated anticoagulation versus antiplatelet treatment in patients with embolic stroke of undetermined source (ESUS) showed that there is no benefit of anticoagulation in the absence of a diagnosis of AF, diagnosing paroxysmal AF continues to be an essential part of the etiological investigation of stroke patients.^1^, ^2^ However, finding paroxysmal AF may be challenging.

It is well known that longer heart rhythm monitoring is associated with a higher probability of detecting paroxysmal AF and the longer the duration of monitoring the higher is the probability of actually finding AF.^3^ However, performing long term heart rhythm monitoring with insertable cardiac monitors in all stroke patients is currently not feasible due to resources constrains. Therefore, an effective pre‐selection of patients with a higher likelihood of having AF would be very useful in clinical practice. In the last years, several studies reported biomarkers associated with an increased risk of developing AF. These biomarkers include morphological cardiac features (atrial fibrosis, left atrial volume,^4^ blood velocity in the left atrial appendage^5^), electrocardiographic characteristics (P‐wave duration and morphology^6^) and blood analytical determinations (NT‐proBNP, MR‐proANP^7^, ^8^). Evidence suggests that these biomarkers may actually be features of an atrial cardiopathy characterized by morphological and structural changes in the atria with associated remodeling that ultimately contributes to the development of AF.^9^ Atrial cardiopathy has even been associated with stroke risk in the absence of AF.^9^

In the current study, the authors evaluated if a cardiac morphological parameter, periatrial epicardial adipose tissue thickness (pEATT), could be used as a new potential biomarker of AF‐associated stroke.^10^ pEATT has previously been shown to be associated with the presence of AF, its severity, and recurrence in patients in general.^11^ pEATT may contribute to AF due to the secretion of inflammatory mediators, increased oxidation stress, and promotion of fibrosis with consequent disruption of the heart conduction system.^11^ In the current study, Edsen et al. retrospectively included 121 patients with AF‐related acute stroke and 94 patients with acute non‐cardioembolic stroke with large vessels occlusion that had been prospectively consecutively collected.^10^ pEATT was evaluated using data from the admission computed tomographic angiographies that routinely included the supra‐aortic vessels as well as the heart. After data analysis, patients with AF‐related stroke were shown to have increased right and left sided pEATT. In a multivariate analysis adjusted for potential confounders including coronary heart diseases, age, body mass index, left atrial enlargement and NT‐proBNP, left sided pEATT was found to be an independent marker of AF‐related stroke. This study has evident limitations like a retrospective design, a small sample size, a lack of external validation of the results, and an absence of evaluation of the biomarker in patients with cryptogenic stroke. Nevertheless, it adds one more piece of data that may help us to further improve our knowledge of cardioembolic stroke. A better comprehension of the features associated with cardioembolic stroke may help to refine the selection of stroke patients that are more likely to have AF.

Still, some steps have to be taken, prior to the use of these biomarkers in clinical practice. In the future, it would be interesting to know which biomarkers or combinations of biomarkers have the highest accuracy for the diagnosis of AF. Also, currently, there is weak evidence of a useful role for blood, ECG, and brain imaging biomarkers for the identification of patients at high risk of AF mainly because there is a lack of clinical trials evaluating their effectiveness to select patients that may benefit the most from AF monitoring.^3^ A pioneer clinical trial (The AtRial Cardiopathy and Antithrombotic Drugs In prevention After cryptogenic stroke randomized trial—ARCADIA) is suppressing this step of searching for AF and directly using biomarkers that have been associated with atrial cardiomyopathy (NT‐proBNP, P‐wave terminal force >5000 μV × ms in ECG lead V_1_, serum NT‐proBNP >250 pg/mL, and left atrial diameter index ≥3 cm/m^2^ on echocardiogram) to select patients to test if anticoagulant therapy reduces stroke recurrence in patients with atrial cardiopathy but no known AF.^12^ This is a clear example of how precision medicine can be used to improve the selection of patients that may benefit the most from certain treatments.

Step by step, we are getting closer to better identify patients with AF‐associated stroke and therefore to be able to provide these patients with the best prevention treatment to reduce stroke recurrence.

## References

[1] Diener HC , Sacco RL , Easton JD , et al. Dabigatran for prevention of stroke after embolic stroke of undetermined source. N Engl J Med. 2019;380(20):1906‐1917. 31091372 10.1056/NEJMoa1813959

[2] Hart RG , Sharma M , Mundl H , et al. Rivaroxaban for stroke prevention after embolic stroke of undetermined source. N Engl J Med. 2018;378(23):2191‐2201. 29766772 10.1056/NEJMoa1802686

[3] Rubiera M , Aires A , Antonenko K , et al. European Stroke Organisation (ESO) guideline on screening for subclinical atrial fibrillation after stroke or transient ischaemic attack of undetermined origin. Eur Stroke J. 2022;239698732210994. 10.1177/23969873221099478 PMC9446336 36082257

[4] Fonseca AC , Alves P , Inácio N , et al. Patients with undetermined stroke have increased atrial fibrosis: a cardiac magnetic resonance imaging study. Stroke. 2018;49:734‐737. 29371431 10.1161/STROKEAHA.117.019641

[5] Goldman ME , Pearce LA , Hart RG , et al. Pathophysiologic correlates of thromboembolism in nonvalvular atrial fibrillation: I. reduced flow velocity in the left atrial appendage (the stroke prevention in atrial fibrillation [SPAF‐III] study). J Am Soc Echocardiogr. 1999;12:1080‐1087. 10588784 10.1016/s0894-7317(99)70105-7

[6] Skrebelyte‐Strøm L , Rønning OM , Dahl FA , Steine K , Kjekshus H . Prediction of occult atrial fibrillation in patients after cryptogenic stroke and transient ischaemic attack: PROACTIA. Europace. 2022:euac092. 10.1093/europace/euac092 35819199 PMC9733955

[7] Fonseca AC , Brito D , Pinho E , et al. N‐terminal pro‐brain natriuretic peptide shows diagnostic accuracy for detecting atrial fibrillation in cryptogenic stroke patients. Int J Stroke. 2014;9:419‐425. 23981586 10.1111/ijs.12126

[8] Schweizer J , Arnold M , König IR , et al. Measurement of Midregional pro‐atrial natriuretic peptide to discover atrial fibrillation in patients with ischemic stroke. J Am Coll Cardiol. 2022;79:1369‐1381. 35393018 10.1016/j.jacc.2022.01.042

[9] Kreimer F , Gotzmann M . Left atrial cardiomyopathy—a challenging diagnosis. Front Cardiovasc Med. 2022;9:942385. 35845077 10.3389/fcvm.2022.942385 PMC9280085

[10] Edsen F , Habib P , Matz O , et al. Epicardial adipose tissue thickness assessed by CT is a marker of atrial fibrillation in stroke patients. Ann Clin Transl Neurol. 2022. 10.1002/acn3.51617 PMC9539378 36191057

[11] Wong CX , Ganesan AN , Selvanayagam JB . Epicardial fat and atrial fibrillation: current evidence, potential mechanisms, clinical implications, and future directions. Eur Heart J. 2017;38(17):1294‐1302. 26935271 10.1093/eurheartj/ehw045

[12] Kamel H , Longstreth WT Jr , Tirschwell DL , et al. The AtRial cardiopathy and antithrombotic drugs in prevention after cryptogenic stroke randomized trial: rationale and methods. Int J Stroke. 2019;14(2):207‐214. 30196789 10.1177/1747493018799981 PMC6645380

